# A new paramutation-like example at the *Delta* gene of *Drosophila*

**DOI:** 10.1371/journal.pone.0172780

**Published:** 2017-03-29

**Authors:** Maria Capovilla, Alain Robichon, Minoo Rassoulzadegan

**Affiliations:** 1 UMR 1355–7254 INRA/Université Côte d’Azur/CNRS, Institut Sophia Agrobiotech, 400 route des Chappes, Sophia Antipolis, France; 2 Université Côte d’Azur, Inserm U1091–CNRS U7277, Nice, France; Biomedical Sciences Research Center Alexander Fleming, GREECE

## Abstract

The hereditary transmission of a phenotype independent from DNA sequence implies epigenetic effects. Paramutation is a heritable epigenetic phenomenon observed in plants and animals. To investigate paramutation in *Drosophila*, we used the *P{ry*^*+t7*.*2*^ *= PZ}Dl*^*05151*^ P-element insertion in the *Drosophila melanogaster* genome that causes a dominant visible phenotype: the presence of characteristic extra-veins in the fly wings. This extra-vein phenotype presents variable expressivity and incomplete penetrance. The insert is a PZ element located 680 bp upstream from the ATG of the *Delta* (*Dl*) gene, encoding the Notch ligand involved in wing vein development, and acts as a null allele. In the G2 offspring from a cross between the heterozygous transgenic stock and wild-type flies, we observed the transmission of the extra-vein phenotype to wild-type flies without the transgene, independently of gender and across many generations. This is a “paramutation-like” example in the fly: the heritable transmission of a phenotypic change not linked to a classical genetic mutation. A “paramutagenic” allele in heterozygotes transmits the phenotype of the heterozygotes to the wild-type allele (“paramutant”) in a stable manner through generations. Distinct from paramutation events so far described in *Drosophila*, here we deal with a dominant effect on a single gene involving variable hereditary signals.

## Introduction

Changes in gene expression arise because of genetic and environmental variation in living organisms [1]. The extent to which these changes are maintained long enough to be transmitted to the next generation is still an open question. Analysis of model organisms largely highlights epigenetic determinants involved in this process.

Paramutation is a classical case of genetically-driven heritable epigenetic variation ([2] and references therein). Pioneer cases of plant paramutation refer to changes in the expressivity of alleles originating from heterozygotes (reviewed in [3–5]). Subsequently, the modified phenotype can be stably propagated in a number of generations. It is dependent upon RNA metabolism, even though, in the case of the maize *booster 1* (*b1*) locus, DNA methylation of an upstream repeated motif is associated to the establishment of paramutation (reviewed in [4–7]). In addition to plants, several cases of paramutation events are described in laboratory mouse [8,9]. A different case of a white tail tip phenotype was observed in wild-type animals for the *Kit* locus, coming from heterozygous *Kit*^*m1Alf*^/*Kit*^*+*^ mice, carrying the *Kit*^*m1Alf*^ *lacZ* insertion [10]. This phenotype is efficiently transmitted and results from a decrease in *c-Kit* messenger RNA levels with the accumulation of non-polyadenylated RNA molecules. A heritable white tail tip phenotype is also observed after microinjection into fertilized eggs of either total RNA from *Kit*^*m1Alf*^*/Kit*^*+*^ mice or fragments of *c-Kit* and cognate microRNAs [10]. More phenotypes were reported by microinjection of other microRNAs altering cellular size of the hearth and growth rate of the stem cells [11,12]. These are all cases of “paramutation-like” phenomena, the heritable transmission of a phenotypic change not linked to a classical genetic mutation and carried out by small noncoding RNAs.

Another case of transcriptional variation is transvection, initially discovered in the fly through somatic cell studies of the *bithorax* complex [13] and later in *Neurospora crassa* [14]. It is a pairing-dependent interaction between alleles (reviewed in [15,16]). More recently, in *Drosophila*, paramutation events involving the transmission of small non coding RNAs (piRNAs) with important silencing and maternal effects were associated with the transmission of a phenotype over 100 generations [17–19]). siRNA silencing discovered in worms and plants is transmissible to further generations with some degrees of reversibility [20]. Finally, in laboratory worms, genetic-mediated variation of siRNAs promotes gene expression alterations and phenotypic changes, which transmission is correlated with heredity of the modified phenotype (reviewed in [21,22]).

In order to find a *bona fide* case of paramutation in *Drosophila*, we have searched for a dominant phenotype caused by a transgene in this model system. We report that the transgene *P{ry*^*+t7*.*2*^ *= PZ}Dl*^*05151*^ (hereafter called *Dl*^*05151*^) in the *Delta* (*Dl*) locus of *Drosophila* [23] produces a dominant extra-vein phenotype in the wing, especially around the posterior cross vein (pcv). The *Drosophila* wing is formed by ventral and dorsal epithelia sheets apposed to each other that are supported by longitudinal and transversal thickenings called veins that function as vessels hosting trachea, hemolymph, nerves and blood cells. *Drosophila* wing vein development is orchestrated by a number of transcription factors and signaling molecules (reviewed in [24]). *Notch* (*N*), together with its ligand *Dl*, restricts vein thickness [25–29].

Starting from heterozygotes, the extra-vein phenotype could be followed in further generations even in the progenies that did not inherit the transgene. The *Dl*^*05151*^ allele acts as a paramutagenic allele that converts the wild-type allele to the parental state. This system provides a powerful model to study paramutation in *Drosophila*, a system almost lacking DNA methylation [30–33]. Overall, our data indicate that an epigenetically-regulated phenotype could be derived from initial genetic events.

## Results and discussion

**Fig 1:**
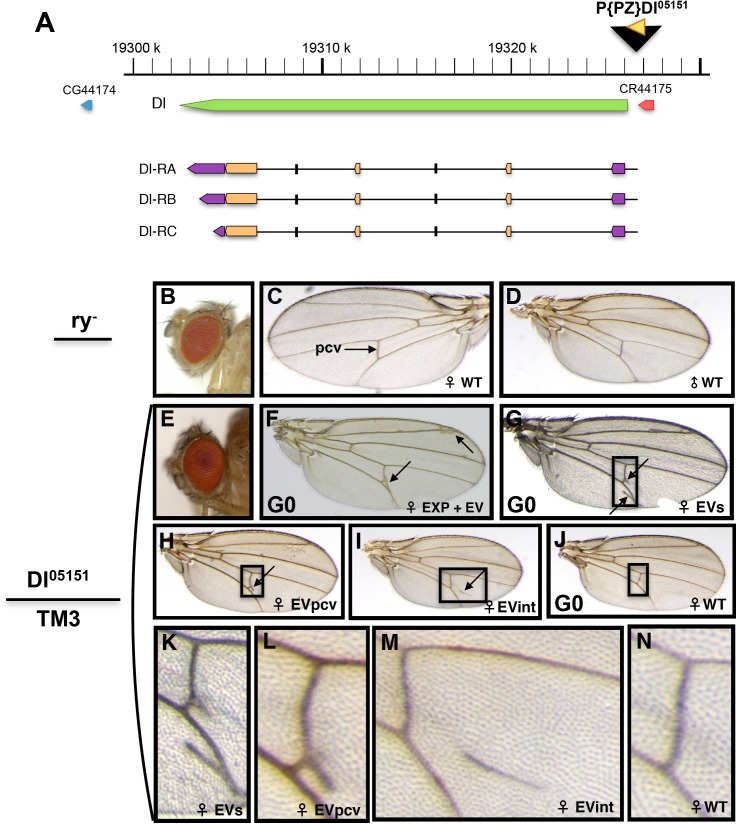
Map of the *Dl* gene and phenotype of *Dl*^*05151*^*/TM3* wings. (A) The *Dl* gene is located at cytological position 92A1-92A2 and between nucleotides 19,302,731 and 19,326,217 of the 3R arm. It has three annotated transcripts transcribed from right to left and is flanked by transcripts CG44174 and CR44175. Green: gene extent of *Dl*. Beige: translated exons. Purple: transcribed exons. Horizontal black lines: introns. This map is extracted from Release R6.08 of the *Drosophila* genome sequence in FlyBase. The *Dl*^*05151*^ insertion (black triangle) is located 681 bp upstream from the *Dl* ATG and is transcribed from right to left. (B) Eye of a *ry*^*-*^*/ry*^*-*^ fly. (C) Left wing of a *ry*^*-*^*/ry*^*-*^ female. (D) Right wing of a *ry*^*-*^*/ry*^*-*^ male. (E) Eye of a heterozygous *Dl*^*05151*^*/TM3* fly. The transgene carrying the *ry*^*+*^ gene, the eye color is as in wild-type. (F) Right wing of a *Dl*^*05151*^*/TM3* female. Arrows point to expanded and extra-veins (EXP+EV). (G) Right wing of a *Dl*^*05151*^*/TM3* female presenting two extra-veins (EVs). (H) Wing of a *Dl*^*05151*^*/TM3* heterozygous female presenting one extra-vein perpendicular to the posterior cross vein (pcv) (EVpcv). (I) Example of an extra-vein in the L4-L5 inter-vein region (EVint). (J) Wild-type wing. Hereafter, wing orientations are according to their origin: left wings with distal tip to the left and right wings with distal tip to the right.

*P{ry*^*+t7*.*2*^ *= PZ}Dl*^*05151*^ (hereafter *Dl*^*05151*^) is the insertion of a pP{PZ} element 681 base pairs upstream from the ATG of the *Delta* (*Dl*) gene of *Drosophila* (http://flybase.org/reports/FBti0004778.html; [34]) (Fig 1A). The pP{PZ} transgene (http://flybase.org/reports/FBmc0002678.html) contains the *lacZ* coding region directed by the Hsp70 promoter, the *rosy* (*ry*) eye marker and plasmid sequences [35]. *Dl*^*05151*^ is an excellent example of enhancer trapping, the random insertion of reporter vectors in the genome of *Drosophila*. Its direction of transcription (Fig 1A) and pattern of *lacZ* expression (S1 Fig) are as for the *Dl* gene [36,37]. In the *Drosophila* developing wing, *Dl* is more highly expressed within the wing veins, while *Notch* (*N*) is more highly expressed in regions between the wing veins [29,37,38].

The *Dl*^*05151*^ transgene in the *Dl* gene of *Drosophila* has been inserted in the *ry*^*506*^ (hereafter *ry*^*-*^*)* background ([23]; Fig 1B). Fig 1C and 1D show examples of wild-type *ry*^*-*^*/ry*^*-*^ wings. As *Dl*^*05151*^ carries the *ry* marker, the flies carrying it have ry^+^ eyes (Fig 1E). *Dl*^*05151*^ causes a dominant phenotype: expanded and extra-veins as shown by *Dl*^*05151*^*/TM3*, *ry*^*RK*^, *Sb* (hereafter *TM3*) flies (Fig 1F–1I). This phenotype displays highly variable expressivity including: extra-veins (EV) and expanded veins (EXP; Fig 1F), two EVs emerging from the posterior cross vein (pcv) and from L5 going anteriorly and posteriorly (EVs; Fig 1G and 1K), one EV perpendicular to the pcv (EVpcv) going posteriorly (Fig 1H and 1L), and an EV in the L4/L5 inter-vein region (EVint; Fig 1I and 1M). However, few wild-type wings are also observed (Fig 1J and 1N). The penetrance is higher in females than in males, but incomplete in both (S1 Table). This insertion behaves as a null *Dl* allele being homozygous lethal and when heterozygous with either the *Dl*^*X*^ [39] or the *Dl*^*RevF10*^ [40] *Dl* amorph alleles. Indeed, the phenotypes of heterozygous *Dl*^*X*^ and *Dl*^*RevF10*^ (S2 Fig) are extra-veins very similar to those of the *Dl*^*05151*^ heterozygous flies (Fig 1), indicating that the extra-vein phenotype of *Dl*^*05151*^ heterozygotes is due to a strong decrease in *Dl* expression.

**Fig 2:**
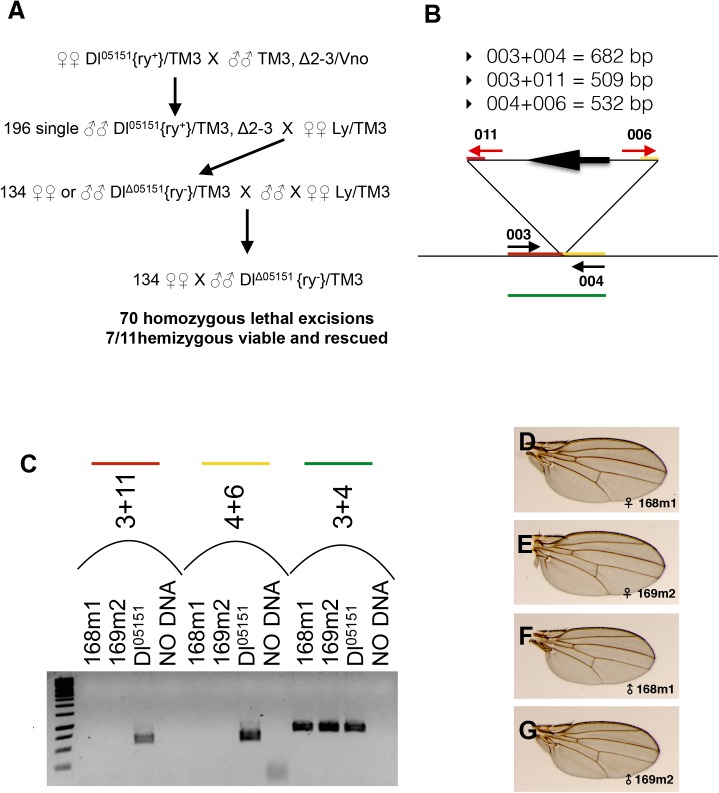
Perfect excision of the *Dl*^*05151*^ insert. (A) Crosses carried out to obtain perfect excisions of the *Dl*^*05151*^ transgene. *Δ2–3* is the chromosome carrying the transposase, *Vno* and *Ly* are dominant wing markers, {ry^+^} indicates that the flies have ry^+^ eyes because they carry the *Dl*^*05151*^ insertion, while {ry^-^} indicates that the flies have ry^-^ eyes because they are devoid of the *Dl*^*05151*^ transgene. *Dl*^*Δ05151*^ are the chromosomes in which an excision of *Dl*^*05151*^ occurred. At least seven excised hemizygous viable and rescued stocks we obtained. (B) Sizes and map of the amplicons obtained by PCR analysis. Oligonucleotides 003 and 004 are downstream and upstream of the *Dl*^*05151*^ transgene, respectively. Oligonucleotides 006 and 011 are in the 5’ and 3’ of the transgene, respectively. (C) 168m1 and 169m2 are two independent hemizygous viable *ry*^*-*^ lines in which excision of *Dl*^*05151*^ occurred through Δ2–3 transposase-mediated excision. As shown by the absence of amplification with the couples of oligonucleotides at the 3’ (red line) and 5’ (yellow line) ends of the transgene, these two strains do not carry the transgene. Sequencing of the *Dl* amplicons (green line) shows that the excisions are perfect. (D-G) Wild-type wings of *Dl*^*Δ05151*^*/ry*^*-*^ females for the two stocks 168m1 and 169m2 as indicated.

The extra-vein phenotype of heterozygous *Dl*^*05151*^ flies being due solely to the P{ry^+t7.2^] = PZ}Dl^05151^ insertion was confirmed through the perfect excision of the transgene by crossing *Dl*^*05151*^*/TM3*, *ry*^*RK*^, *Sb* (hereafter *TM3*) females with males carrying the Δ2–3 transposase [41] (see Fig 2A for crosses and Materials and Methods for details). One hundred and ninety-six *Dl*^*05151*^*/P{ry*^*[+t7*.*2*^ *= Delta2-3}99B* males were crossed individually to *Ly/TM3*, *ry*^*-*^, *Sb* females and 134 excisions over *TM3*, *ry*^*-*^, *Sb* stocks were established and analyzed. Seventy independent excisions were obtained and they were all homozygous lethal. To test whether the excised chromosomes carry a secondary lethal, 11 excised stocks were crossed to the *Dl Df(3R)BSC850* deficiency. Of these 11 stocks, seven were viable when hemizygous against the *Dl* deficiency. Using the oligonucleotides shown in Fig 2B, we get evidence that at least two out of these seven (168m1 and 169m2) do not carry the *Dl*^*05151*^ insert (Fig 2C) and display a normal sequence. All the seven perfectly excised lines present a full rescue of the dominant wing phenotype after outcrossing to *ry*^*-*^ (Fig 2D–2G). Thus, the chromosome carrying the *Dl*^*05151*^ insert bears a secondary lethal and clearly the *Dl*^*05151*^ insertion is the cause of the extra-vein phenotype (Fig 1).

**Fig 3:**
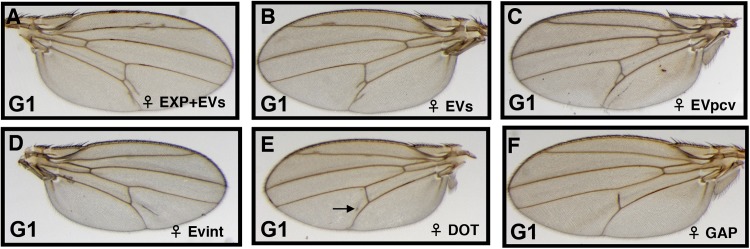
Wing phenotypes of heterozygous *Dl*^*05151*^/Dl* females. Wings of the *Dl*^*05151*^*/Dl*^*+*^ progeny of an out cross of *Dl*^*05151*^*/TM3* males to *ry*^*-*^*/ry*^*-*^ females. A whole spectrum of extra-vein phenotypes is found. (A) Expanded and extra-veins (EXP+EVs). (B) Two extra-veins (EVs). (C) One extra-vein emerging from the pcv (EVpcv). (D) An extra-vein in the inter-vein region (EVint). (E) A dot in the inter-vein region (arrow; DOT). (F) An anterior gap of the pcv (GAP). The spectrum of phenotypes is very similar to that of the *Dl*^*05151*^*/TM3* stock.

To investigate a paramutation event associated with the *Dl*^*05151*^ insert, five *Dl*^*05151*^/*TM3* heterozygous male*s* were individually crossed to homozygous *ry*^*-*^*/ry*^*-*^ flies. The wings of first generation (G1) *Dl*^*05151*^, *ry*^*-*^/Dl*, *ry*^*-*^ (hereafter, *Dl*^*05151*^/Dl*; where Dl* indicates the *ry* chromosome that is in heterozygosis with *Dl*^*05151*^ in the G1) heterozygotes present an extra-vein phenotype with highly variable expressivity as in the parental *Dl*^*05151*^/*TM3* stock (Fig 3). Occasionally, the pcv presents a gap (GAP; Fig 3F). The phenotype is dominant and G1 flies show incomplete penetrance as for the parental *Dl*^*05151*^/*TM3* stock (Table 1).

**Table 1:** Quantification of the extra-vein phenotypes in the G0, G1 and G2.

	% EV ± SE				χ^2^ (df = 1)	
GENOTYPE	♀		♂		♀	♂
Dl^05151^/TM3, Sb G0	85.11	± 2.32	40.30	± 2.7	456.90 (p< 0.001)	155.67 (p< 0.001)
Dl^05151^/Dl^+^ G1	97.49	± 1.77	51.56	± 6.25	398.21 (p< 0.001)	148.29 (p< 0.001)
Dl^05151^/Dl^+^ G2	94.05	± 1.13	51.03	± 2.39	701.52 (p< 0.001)	233.19 (p< 0.001)
Dl*/Dl^+^ G2 from ♀ G1	26.45	± 3.54	28.97	± 3.77	75.02 (p< 0.001)	81.17 (p< 0.001)
Dl*/Dl^+^ G2 from ♂ G1	9.01	± 1.88	13.52	± 2.19	12.32 (p< 0.001)	27.50 (p< 0.001)
ry^-^/ry^-^ stock	2.47	± 0.77	2.38	± 0.78		

Females and males were counted separately. G0 are the founder *Dl*^*05151*^*/TM3* flies, G1 are the progenies of the cross of the founder males to *ry*^**-**^ females and G2 are the progenies of the back cross of G1 females and males to *ry*^**-**^ flies. At the bottom, is the quantification of the phenotype in the *ry*^*-*^*/ry*^*-*^ host. Dl* indicates the chromosome that has been in contact with the transgene in the G1. EV: presence of at least one extra-vein. % EV: percent of extra-veins. The standard deviation (SE) and χ^2^ are indicated.

**Fig 4:**
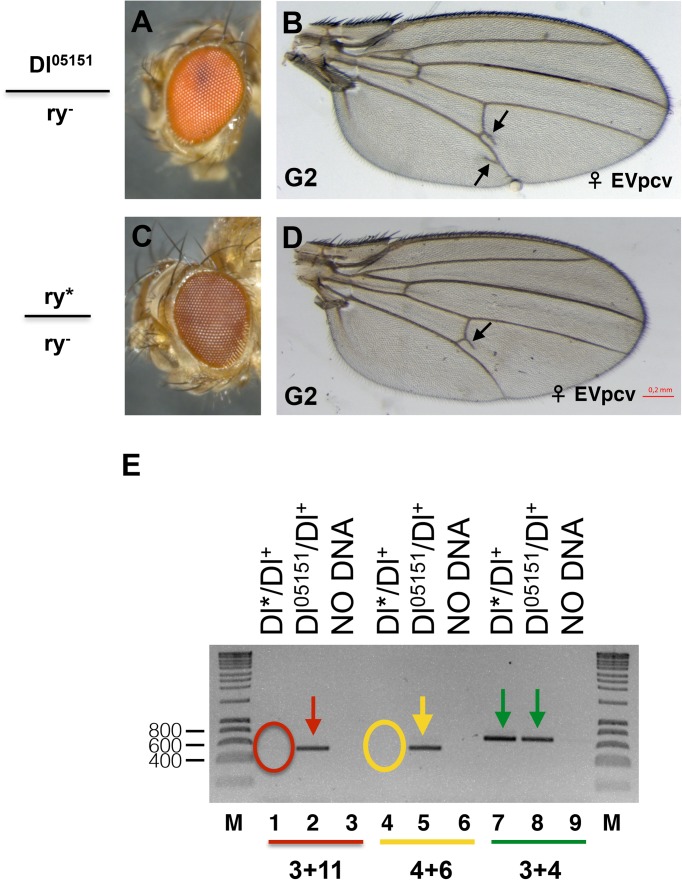
Paramutation at the *Dl* locus. Eye (A) and wing (B) of a heterozygous G2 *Dl*^*05151*^/*Dl*^*+*^ fly. The wing shown presents a frequent phenotype: two extra-veins near the pcv (arrows). Eye (C) and wing (D) of a *Dl*^*05151*^, *ry*^*-*^/Dl*, *ry*^*-*^ (hereafter, *Dl*^*05151*^/Dl*) fly. This G2 fly does not carry the *Dl*^*05151*^ insert, but shows an extra-vein perpendicular to the pcv (arrow) indicating that its *Dl*^*+*^ (Dl*) chromosome coming from the heterozygous *Dl*^*05151*^/Dl* parent has been paramutated. (E) PCR on the genomic DNA of the fly carrying the wing shown in Fig 4D. The oligonucleotides used are shown in Fig 2B. In the fly carrying the transgene (*Dl*^*05151*^*/Dl*^*+*^), oligonucleotides 003 and 011 (red arrow) and 004 and 006 (yellow arrow) amplify the 3’ and 5’ ends of the transgene, respectively. No amplification with these two couples of primers is seen for the paramutant Dl*/Dl^*+*^ fly (red and yellow circles, respectively). Control amplification with oligonucleotides 003 and 004 (green arrows) in the *Dl* gene occurs for both flies.

**Fig 5:**
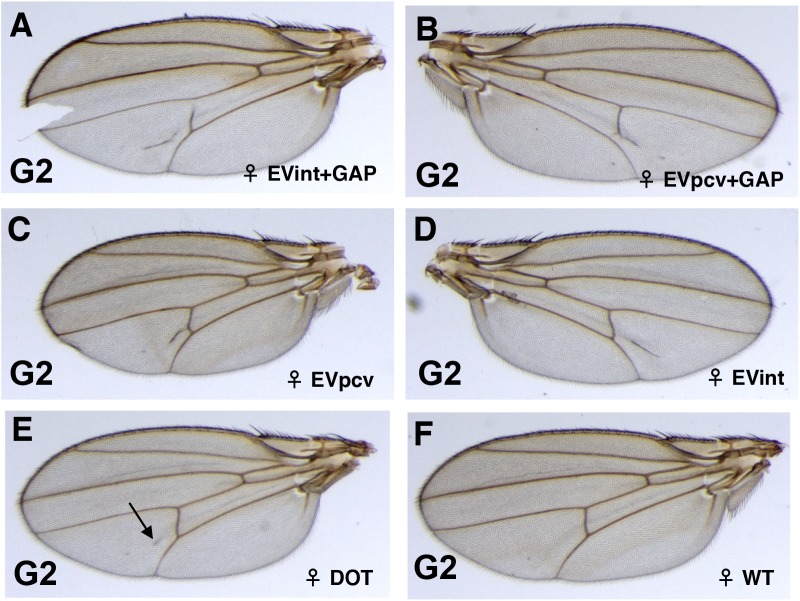
Variability in the expressivity of the extra-vein phenotypes in the wings of the G2 Dl*/Dl^*+*^ flies. In the G2, paramutant Dl*/Dl^*+*^ flies exhibit a wide range of wing vein phenotypes. (A) An extra-vein in the inter-vein region together with a pcv gap (EVint+GAP). (B) An extra-vein perpendicular to the pcv together with a pcv gap (EVpcv+GAP). (C) An extra-vein perpendicular to the pcv (EVpcv), (D) An extra-vein in the inter-vein region (EVint), (E) A faint dot (arrow; DOT). (F) A percentage of wings are wild-type (WT).

In order to obtain wild-type flies without the *Dl*^*05151*^ insertion, G1 *Dl*^*05151*^/Dl* females (Fig 4A and 4B) and males where crossed in mass to homozygous *ry*^*-*^*/ry*^*-*^ flies (see S4 Fig for crosses). In the G2 progeny, it was observed a variable percentage of Dl*, *ry*^*-*^*/Dl*^+^, *ry*^*-*^ (where *Dl*^+^ indicates the *ry*^*-*^ chromosome wild-type for *Dl*; hereafter Dl*/Dl^*+*^) flies with different degrees of extra-veins (Figs 4D and 5). We tested by PCR whether these paramutant flies carry the *Dl*^*05151*^ transgene, using the same oligonucleotide combinations as described above (Fig 2B). A representative PCR experiment for the fly shown in Fig 4D clearly demonstrates that this fly does not carry the *Dl*^*05151*^ transgene (Fig 4E, lanes 1 and 4). Fig 5 shows examples of the observed phenotypes of Dl*/Dl^*+*^ females including gaps (Fig 5A and 5B), extra-veins perpendicular to the pcv (Fig 5B and 5C), extra-veins in the inter-vein region (Fig 5A and 5D) and small dots (Fig 5E). This weak phenotype and extra-veins in the L4/L5 inter-vein region are also observed in the *ry*^*-*^*/ry*^*-*^ stock at a considerably lower frequency (Table 1; S3 Fig). Many wild-type flies are also found (Fig 5F). The penetrance rate of the extra-vein phenotype in the total G2 Dl*/ Dl^*+*^ wings is higher than in the wings of the *ry*^*-*^*/ry*^*-*^ background and this difference is highly significant (Table 1). The penetrance rate is significantly higher in the offspring of G1 *Dl*^*05151*^/Dl* females compared to the offspring of G1 *Dl*^*05151*^/Dl* males (χ^2^ = 19.8; df = 1; p<0.001 and χ^2^ = 12.96; df = 1; p<0.001 for females and males, respectively), possibly because the phenotype is more penetrant in females than in males carrying the insert. Detailed data for each individual male are reported in S2 Table.

The *ry*^*-*^ chromosome that has been in contact with the *Dl*^*05151*^ transgene (indicated as Dl*) has been “paramutated”, leading to the same phenotype as that caused by the insertion. This result is reminiscent of paramutation at the mouse *Kit* locus [10], where homozygous G2 wild-type mice from *Kit*^*tm1Alf/+*^ heterozygotes exhibited the white feet and white tail phenotype of heterozygous parents carrying a *lacZ* insertion in the *Kit* locus (*Kit*^*tm1Alf*^). Both events call for the transmission of a visible character through epigenetic mechanisms modifying gene expression.

**Fig 6:**
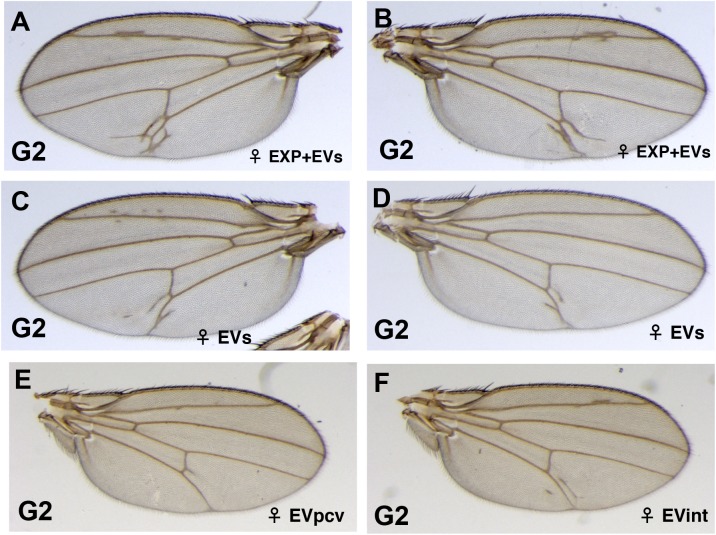
Strong expressivity of the extra-vein phenotype in the heterozygous G2 *Dl*^*05151*^/*Dl*^*+*^ flies. In the G2, *Dl*^*05151*^/*Dl*^*+*^ flies present wings with many extra-veins. The phenotype ranges from two or more extra-veins of various lengths (A-D), to one extra vein perpendicular to the pcv (E), to an extra-vein in the inter-vein region (F).

It is worth noticing that the *Dl*^*05151*^*/Dl*^*+*^ G2 ry^+^ offspring carrying the *Dl*^*05151*^ insertion shows a stronger phenotype than its parents (Fig 1). Often, many extra veins are observed (Fig 6A–6D). Other frequent phenotypes are the EVpcv (Fig 6E) and the EVint (Fig 6F). This shows that the phenotype segregates with the insertion and suggests that the paramutagenic allele continues to produce signals that amplify the phenotype.

We tested whether paramutation requires pairing through balancer chromosomes, which prevent pairing, as they are rich in inversions. Paramutation likely requires pairing as the extra-vein phenotype is not transmitted to the *TM3* balancer chromosome in the G1 and is rarely transmitted to the *TM2*, *ry* balancer (S3 Table). Nevertheless, it is also possible that balancer chromosomes carry suppressors that mask the phenotype.

To assess a possible transgenerational effect, the *TM2*, *ry* balancer was used to mark the Dl* chromosome in the G3. G2 females and males Dl*/TM2, *ry* were crossed to each other and the wing phenotypes assessed in the G3 offspring (Table 2). The wing phenotype observed was predominantly the weak one of dots, although a few pcv and inter-vein extra veins were observed, especially in Dl*/Dl* homozygotes. Overall, the rate of wing defects in Dl*/TM2 and Dl*/Dl* flies is significantly higher than in the *ry*^*-*^*/ry*^*-*^ background (Table 2) and higher than in the G2 (compare Table 2 with Table 1). This shows that paramutation is carried at least to the third generation.

**Table 2:** Quantification of the extra-vein phenotypes in the G3.

	% EV ± SE				χ^2^ (df = 1)	
GENOTYPE	♀		♂		♀	♂
Dl*/TM2 G3 from ♀ G1	78.05	± 6.46	62.79	± 7.37	240.54 (p< 0.001)	172.53 (p< 0.001)
Dl*/Dl* G3 from ♀ G1	72.73	± 7.75	78.26	± 8.6	200.67 (p< 0.001)	186.88 (p< 0.001)
Dl*/TM2 G3 from ♂ G1	52.50	± 7.9	64.00	± 6.79	132.99 (p< 0.001)	186.52 (p< 0.001)
Dl*/Dl* G3 from ♂ G1	59.38	± 8.68	64.00	± 9.6	145.96 (p< 0.001)	142.61 (p< 0.001)
ry^-^/ry^-^ stock	2.47	± 0.77	2.38	± 0.78		

The Dl* chromosome was marked with the *TM2* balancer. The wings of Dl*/Dl* and Dl*/TM2 flies have a higher percentage of extra-veins than the *ry*^*-*^ background stock. % EV: percent of extra-veins. The standard deviation (SE) and χ^2^ are indicated.

It has long been known that a decrease in *Dl* function leads to thickened veins [25,26]. In our work, it seems that *Dl* is very sensitive to gene expression levels. An even slight decrease in *Dl* expression causes a visible extra-vein phenotype, possibly interfering with the multiple pathways governing wing vein formation [24]. Reduction or up-regulation of several components of these pathways affect wing vein width ranging from no veins to expanded or extra-veins. *N* and *Dl* belong to the “thickened” group of genes affecting lateral inhibition [25] and control vein width possibly through mis-regulation of *rhomboid* (*rho*) [28] and *veinlet* (*ve*) [29]. Interactions of *N* with many other gene products are known [29,42,43]. Our data suggest that the transgene has modified *Dl* expression on the homologous chromosome through a transmissible system that potentiates itself throughout generations producing a very variable wing vein phenotype.

So far, many cases of paramutation and transvection observed in a variety of organisms involve the activity of ncRNAs [44,45]. Direct microinjection into the naive fertilized eggs of total RNAs from the sperm of genetically [10], environmentally [46,47] or metabolically [48,49] stressed animals transfers a phenotype that becomes subsequently hereditary throughout generations. These epigenetic studies are illustrations for the role of ncRNAs in the transmission of information to next generations. Although the direct demonstration of these phenomena in the fly is still missing, our results provide strong evidence for a general type of subtle gene expression variation phenomenon through a paramutation-like process.

**Fig 7:**
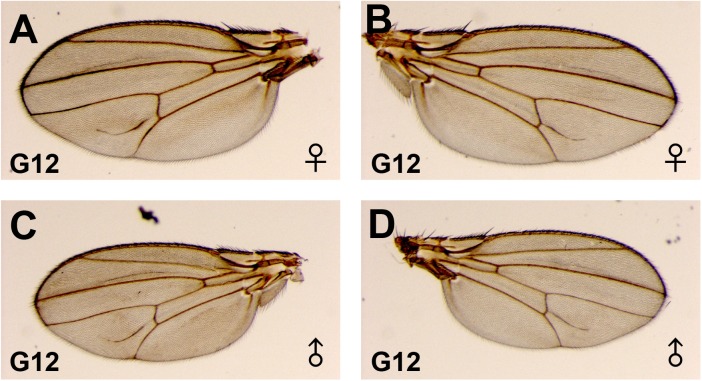
Wing phenotypes in the G12 progeny of the paramutants. In the G12 of inbred Dl*/Dl^*+*^ flies, the extra-vein phenotype showed complete penetrance in both females and males and higher expressivity than in the G2 and G3. In this figure, is shown the most frequent phenotype: a thick extra-vein in the inter-vein region (A-D) sometimes emerging from the pcv (B and D).

To test the capacity of the paramutant Dl* chromosome to become paramutagenic, the extra-vein phenotype was followed through 12 generations of the Dl*/Dl^*+*^ stocks from three independent G0 males. The full penetrance and high expressivity of the paramutant phenotype in the G12 females and males (Fig 7) strongly suggest that the Dl* allele has become stable. The extra-vein of the G12 progeny appears as a real emerging vein (Fig 7B and 7D). This last observation suggests that a paramutant-like phenotype could be selected in an isolated colony of flies recalling conditions for evolution.

It is difficult to extend these observations to humans without a defined genetic example model. However, many human diseases have not yet been linked to genetic causes even by recent genome-wide association studies investigating thousands of genomic markers (reviewed in [50,51]). Transgenerational epigenetic heredity is known to occur in mammals [51–54], affecting gene expression. Mis-regulation of essential genes due to epigenetic causes may be responsible for many human diseases not linked to genetic aberrations. Indeed, some of the missing heritability of human diseases may be explained by the transgenerational transmission of epigenetic information through non-Mendelian inheritance as shown by epigenome-wide association studies [54,55]. The transmission of epigenetic marks from one generation to another through the gametes has been clearly reported so far in model organisms. However in humans, we can find clear genetic aberrations that involve repeats (CGG) and transposable elements [52,56,57] and those seem to rely on DNA methylation and RNAs [58]. An involvement of these players in human diseases and cancer has been recently proposed [52,53,57].

Here we show that the *Drosophila melanogaster* model system, almost devoid of DNA methylation [30,31,33] (a process that has been shown to be associated to paramutation in plants [59]), can be a useful tool to deeply dissect the molecular mechanisms governing non-Mendelian heredity [60].

## Materials and methods

### Crosses and wing analysis

Flies were grown at 23–25°C in standard cornmeal medium with melassa as a source of sugar. The *ry*^*506*^ stock utilized was a kind gift of Anna Digilio (Università di Napoli, Napoli, Italy). The *P{ry*^*+t7*.*2*^ *= PZ}Dl*^*05151*^ stock is Bloomington stock n. 11651. Flies showed a decrease in the expressivity of the extra-vein phenotype through the span of a few years, but the same stocks ordered at different times within the last year show the same variation in penetrance and a high variation in the expressivity of the phenotype. *Df(3R)BSC850* is *w*^*1118*^*; Df(3R)BSC850/TM6C*, *Sb*^*1*^ *cu*^*1*^ (Bloomington stock n. 27922). *Dl*^*X*^ and *Dl*^*RevF10*^ are Bloomington stocks n. 60336 and n. 6300, respectively.

For the phenotypic analysis of the excisions of the P{ry^+t7.2^ = PZ}Dl^05151^ P-element, 20 *Dl*^*05151*^*/TM3*, *ry*^*RK*^, *Sb* females were crossed to ten *y*^*1*^ *w*^*1*^*; Ki*^*1*^ *P{ry*^*+t7*.*2*^ *= Delta2-3}99B* (Bloomington stock n. 4368) males and 134 stocks with ry^-^ eyes (*Dl*^*Δ05151*^*/TM3*, *ry*^*RK*^, *Sb*) were established by crossing individually each *Dl*^*05151*^*/Ki*^*1*^ *P{ry*^*+t7*.*2*^ *= Delta2-3}99B* male to *Ly/TM3*, *ry*^*RK*^, *Sb* females. *Dl*^*Δ05151*^*/TM3*, *ry*^*RK*^, *Sb* stocks were all homozygous lethal. The *Dl* deficiency *Df(3R)BSC850* was used to obtain hemizygous *Dl*^*05151*^-excised animals. Seven out of eleven *Dl*^*Δ05151*^*/Df(3R)BSC850* stocks analyzed were viable. To test for wing rescue, *Dl*^*Δ05151*^*/TM3*, *ry*^*RK*^, *Sb* males were outcrossed to *ry* females and wings of the *Dl*^*Δ05151*^/*ry*^*-*^ progeny analyzed. The *w*^*1118*^*; kar*^*2*^, *ry*^*506*^, *Vno/TM3*, *ry*^*RK*^, *Sb*, *delta2-3* and *Ly*^*1*^, *{FRT80B*, *ry*^*+*^*}*, *kar*^*2*^, *ry*^*506*^ *TM3*, *ry*^*RK*^, Sb stocks were kind gifts of Pascal Heitzler (IBMP, Strasbourg, France).

For the paramutation crosses, five individual *Dl*^*05151*^*/TM3*, *ry*^*RK*^, *Sb* males were crossed to *ry*^*-*^/*ry*^*-*^ females and 1–20 females or males of the G1 *Dl*^*05151*^*/ry*^*-*^ progeny were crossed to *ry*^*-*^/*ry*^*-*^ flies. The G2 progeny analyzed is shown in Table 1 and S2 Table. Penetrance rates of extra-veins in the G2 Dl*/*Dl*^*+*^ and in the *ry*^*-*^/*ry*^*-*^ flies were compared using simple χ^2^ tests. To obtain the G3 balanced with the *TM2*, *ry*^*-*^ balancer, G1 *Dl*^*05151*^/Dl* females and males were crossed to *TM2*, *ry*^*-*^*/MKRS* flies. The resulting G2 Dl*/*TM2*, *ry*^*-*^ females and males were inbred. G1 and G2 data are available upon request.

Wings that looked damaged were discarded. Wings were dissected from the adults using fine Dumont forceps, immersed in 100% Ethanol and mounted in Euparal (BioQuip). Images were acquired thanks to the SPIBOC imaging facility of the Institut Sophia Agrobiotech on a LEICA (Switzerland) MZFLIII stereomicroscope using the ZEN software (ZEISS).

### DNA analysis

DNAs were extracted with phenol and ethanol precipitated. Single fly DNA was resuspended in 20 μl of 10 mM Tris pH7.5, 1 mM EDTA (TE). Ten nanograms of DNA were used for each PCR. For PCR reactions, the MyTaq (Bioline, France) was used in 50 μl reactions according to instructions. Oligonucleotides were used at a 10 mM dilution and their sequences are as follows: 003, 5’-CGGAGTCTTCTCCTTTTCACG-3’; 004, 5’-AAATAAACACCCATCCGGTTGAAG-3’; 006, 5’-TAATAGCACACTTCGGCACG-3’; 011, CAATCATATCGCTGTCTCACTCAG-3’. The cycles were: one minute denaturation at 95°C followed by 35 cycles of 15 sec denaturation at 95°, 15 sec annealing at 57°C and 30 sec extension at 72°C. Reactions ended with 5 min extension at 72°C.

## Supporting information

S1 Fig *lacZ* expression in *Dl*^*05151*^*/TM3* imaginal disks. Imaginal disks of a *Dl*^*05151*^*/TM3* female were stained for *lacZ* expression by X-gal coloration. *lacZ* is expressed in the gut (A), in the brain and in the antenna and eye disk (B), in the wing disk (C), and in the leg disks (D).
(PDF)

S2 Fig Wing phenotypes of *Dl*^*X*^ and *Dl*^*RevF10*^ heterozygous flies. (A) Right wing of a *Dl*^*X*^*/TM3*, *Sb*^*1*^ female. (B) Right wing of a *Dl*^*X*^*/TM3*, *Sb*^*1*^ male. (C) Right wing of a *Dl*^*RevF10*^, e*, *Ser*^*RX82*,^ *P{ry*^*+t7*.*2*^ *= neoFRT}82B/TM6B*, *Tb*^*1*^ female. (D) Right wing of a *Dl*^*RevF10*^, e*, *Ser*^*RX82*,^ *P{ry*^*+t7*.*2*^ *= neoFRT}82B/TM6B*, *Tb*^*1*^ male. These wings show extra-veins very similar to those of *Dl*^*05151*^ heterozygotes (**Figs 1** and **3**).
(PDF)

S3 Fig Weak wing phenotype of the *ry*^*-*^*/ry*^*-*^ background. In a small percentage of flies of the *ry*^*-*^*/ry*^*-*^ stock, extra-veins in the L4/L5 inter-vein region (A) or small dots (B and C) are observed. Many flies have wild-type wings (D).
(PDF)

S4 Fig Crosses carried out to obtain the paramutant flies. (A) Heterozygous *Dl*^*05151*^/*TM3* males were crossed to homozygous *ry*^*-*^*/ry*^*-*^ females and heterozygous *Dl*^*05151*^*/Dl*^*+*^, *ry*^*-*^ females and males were backcrossed to *ry*^*-*^*/ry*^*-*^ flies. In the G2 progeny, a certain number of paramutant Dl*/Dl^*+*^ flies with extra-veins in their wings were found. (B) Heterozygous *Dl*^*05151*^/*TM3* males were crossed to homozygous *ry*^*-*^*/ry*^*-*^ females and heterozygous *Dl*^*05151*^*/ry*^*-*^ females and males were crossed to flies carrying the *TM2* and *MKRS* balancers in order to mark the chromosomes. Dl*/TM2 females and males were crossed to each other and the G3 progeny analyzed.
(PDF)

S1 Table Quantification of the penetrance of the extra-vein phenotype in the *Dl*^*05151*^*/TM3* stock. Amount of females and males with extra-veins in the *Dl*^*05151*^*/TM3* stock. L+R EV: extra veins in both left and right wings. L EV: extra-vein only in the left wing. R EV: extra-vein only in the right wing. WT: absence of extra-veins. TOT: total number of flies. TOT EV: total number of flies with at least one extra-vein. % EV: percent of total extra-veins. Females show higher penetrance and expressivity than males.
(PDF)

S2 Table Detailed quantification of the extra-vein phenotype in the G2. Females and males with extra-veins were counted separately. As shown in the “parents” column, in some cases, more than one cross was carried out with G1 flies showing different phenotypes. The genotype of the counted flies is indicated (GEN.). WT: absence of extra-vein. EV: presence of at least one extra-vein. TOT: total number of flies. % EV: percent of extra-veins. A high variability in the penetrance of the paramutants is observed among crosses, independently from the strength of the phenotype of the parents.
(PDF)

S3 Table Defective wings in flies carrying balancer chromosomes. Females and males carrying the *Dl*^*05151*^ in heterozygosis with the TM3 or TM2 balancers were outcrossed to *ry*^*-*^*/ry*^*-*^ and very few defective wings were found in the progenies containing the balancer chromosomes. WT: wild-type. D: dots. EV: extra-veins. TOT: total number of flies.
(PDF)
