# Supplementary figures and images for: A new paramutation-like example at the Delta gene of Drosophila
Source: PLoS One. 2017 Mar 29;12(3):e0172780. doi: 10.1371/journal.pone.0172780 (PMC5371283; doi:10.1371/journal.pone.0172780)

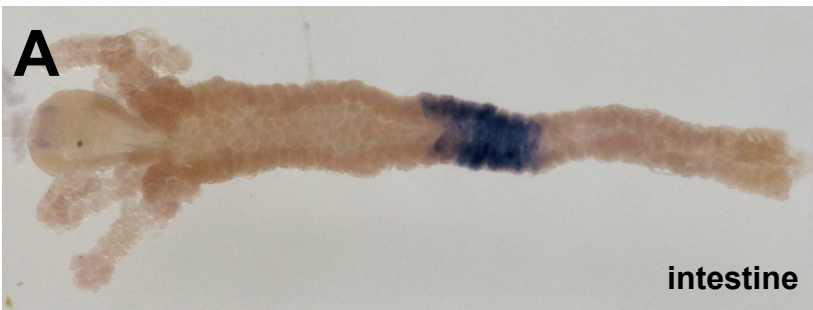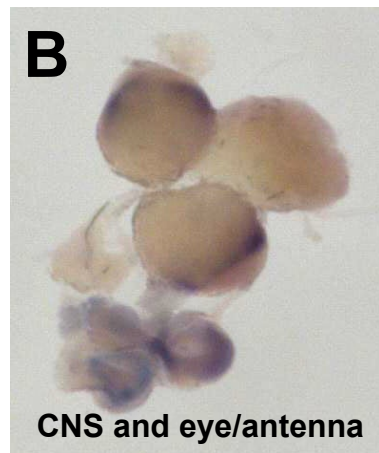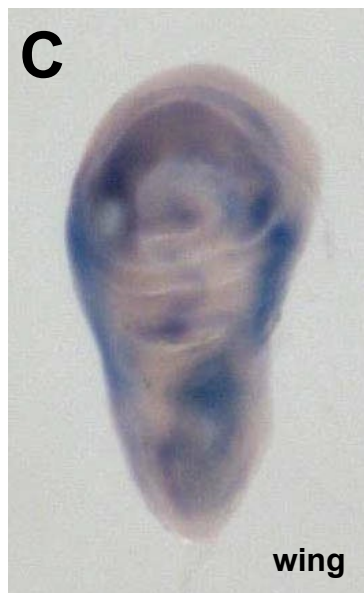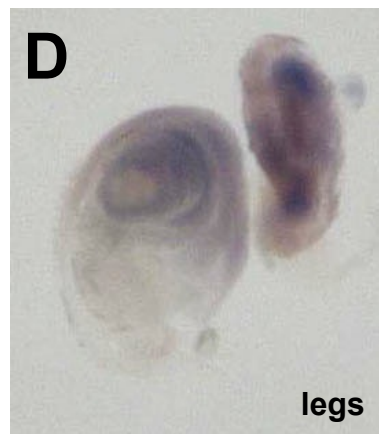

Supplement: S1 Fig — Imaginal disks of a Dl05151/TM3 female were stained for lacZ expression by X-gal coloration. lacZ is expressed in the gut (A), in the brain and in the antenna and eye disk (B), in the wing disk (C), and in the leg disks (D). (PDF) [file pone.0172780.s001.pdf]

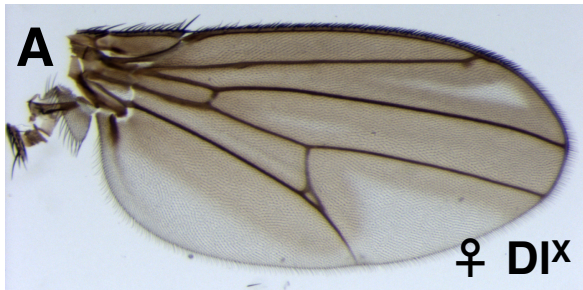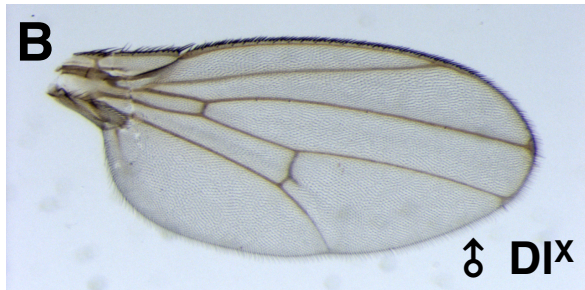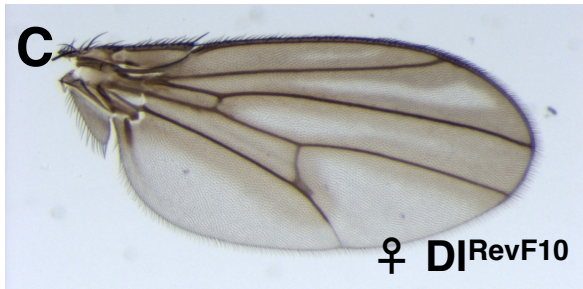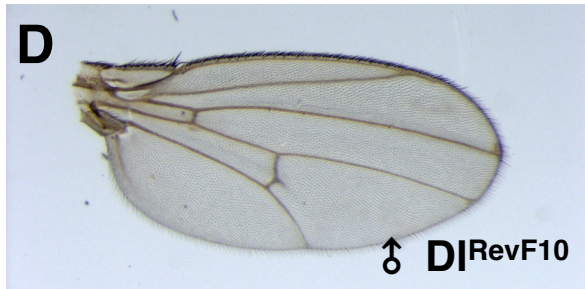

Supplement: S2 Fig — (A) Right wing of a DlX/TM3, Sb1 female. (B) Right wing of a DlX/TM3, Sb1 male. (C) Right wing of a DlRevF10, e*, SerRX82, P{ry+t7.2 = neoFRT}82B/TM6B, Tb1 female. (D) Right wing of a DlRevF10, e*, SerRX82, P{ry+t7.2 = neoFRT}82B/TM6B, Tb1 male. These wings show extra-veins very similar to those of Dl05151 heterozygotes (Figs 1 and 3). (PDF) [file pone.0172780.s002.pdf]

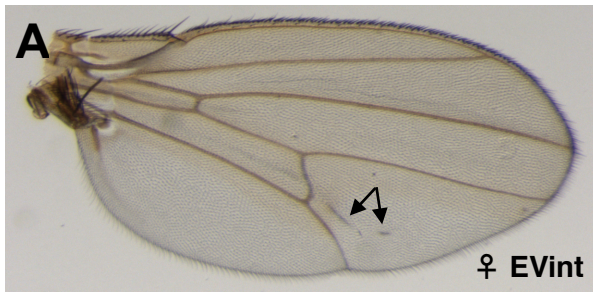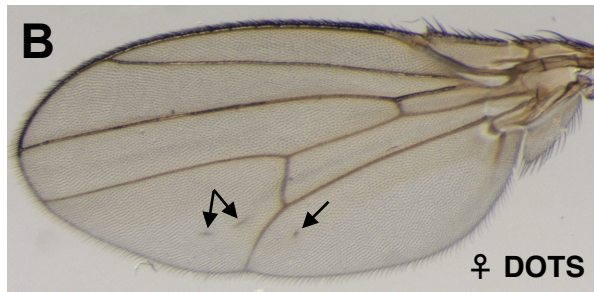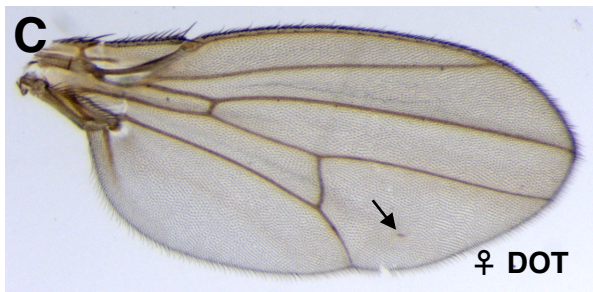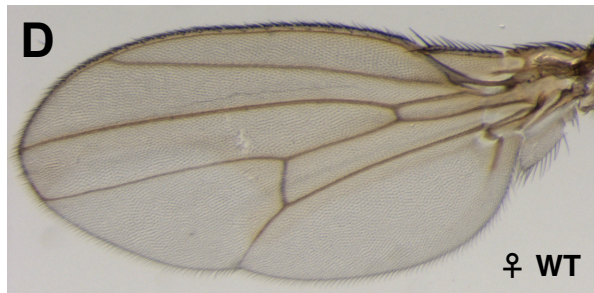

Supplement: S3 Fig — In a small percentage of flies of the ry-/ry- stock, extra-veins in the L4/L5 inter-vein region (A) or small dots (B and C) are observed. Many flies have wild-type wings (D). (PDF) [file pone.0172780.s003.pdf]
