# Supplementary material for: A new paramutation-like example at the Delta gene of Drosophila
Source: PLoS One. 2017 Mar 29;12(3):e0172780. doi: 10.1371/journal.pone.0172780 (PMC5371283; doi:10.1371/journal.pone.0172780)
Supplement: S4 Fig — (A) Heterozygous Dl05151/TM3 males were crossed to homozygous ry-/ry- females and heterozygous Dl05151/Dl+, ry- females and males were backcrossed to ry-/ry- flies. In the G2 progeny, a certain number of paramutant Dl*/Dl+ flies with extra-veins in their wings were found. (B) Heterozygous Dl05151/TM3 males were crossed to homozygous ry-/ry- females and heterozygous Dl05151/ry- females and males were crossed to flies carrying the TM2 and MKRS balancers in order to mark the chromosomes. Dl*/TM2 females and males were crossed to each other and the G3 progeny analyzed. (PDF) [file pone.0172780.s004.pdf]

A

G0: ♂♂ Dl^{05151}/TM3 X ♀♀ Dl^{+}/Dl^{+}

G1: ♀♀ + ♂♂ Dl^{05151}/$\mathbf{Dl}^{*}$ X Dl^{+}/Dl^{+}

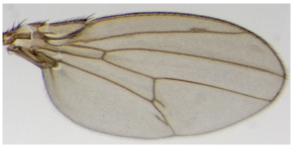

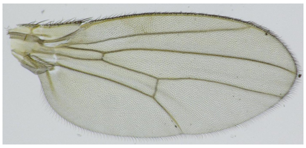

G2: Dl^{05151}/Dl^{+} {ry+} ♀♀ + ♂♂ EV

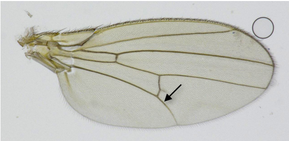

$\mathbf{Dl}^{*}/Dl^{+}$ {ry-} ♀♀ + ♂♂ EV

B

G0: ♂♂ Dl^{05151}/TM3 X ♀♀ Dl^{+}/Dl^{+}

G1: ♀♀ + ♂♂ Dl^{05151}/**Dl*** X ♂♂ + ♀♀ TM2/MKRS

G2: ♀♀ X ♂♂ **Dl***/TM2 {ry+}

G3:

Dl*/TM2 {ry-}

Dl*/**Dl*** {ry-}

♀♀ + ♂♂ EV
