# Supplementary material for: A new paramutation-like example at the Delta gene of Drosophila
Source: PLoS One. 2017 Mar 29;12(3):e0172780. doi: 10.1371/journal.pone.0172780 (PMC5371283; doi:10.1371/journal.pone.0172780)
Supplement: S1 Table — Amount of females and males with extra-veins in the Dl05151/TM3 stock. L+R EV: extra veins in both left and right wings. L EV: extra-vein only in the left wing. R EV: extra-vein only in the right wing. WT: absence of extra-veins. TOT: total number of flies. TOT EV: total number of flies with at least one extra-vein. % EV: percent of total extra-veins. Females show higher penetrance and expressivity than males. (PDF) [file pone.0172780.s005.pdf]

	♀					♂				
	**L+R EV**	**L EV**	**R EV**	**WT**	**TOT**	**L+R EV**	**L EV**	**R EV**	**WT**	**TOT**
	60	20	20	15	115	7	18	11	83	119
**TOT EV**	100					36				
**% EV**	87					30				
