# Supplementary material for: A new paramutation-like example at the Delta gene of Drosophila
Source: PLoS One. 2017 Mar 29;12(3):e0172780. doi: 10.1371/journal.pone.0172780 (PMC5371283; doi:10.1371/journal.pone.0172780)
Supplement: S2 Table — Females and males with extra-veins were counted separately. As shown in the “parents” column, in some cases, more than one cross was carried out with G1 flies showing different phenotypes. The genotype of the counted flies is indicated (GEN.). WT: absence of extra-vein. EV: presence of at least one extra-vein. TOT: total number of flies. % EV: percent of extra-veins. A high variability in the penetrance of the paramutants is observed among crosses, independently from the strength of the phenotype of the parents. (PDF) [file pone.0172780.s006.pdf]

LOG	PARENTS	*Dl^{05151}/Dl^{+}* {ry^{+}}								*Dl^{*}/Dl^{+}* {ry^{-}}							
		♀♀				♂♂				♀♀				♂♂			
		WT	EV		TOT	WT	EV		TOT	WT	EV		TOT	WT	EV		TOT
		n	n	%	n	n	n	%	n	n	n	%	n	n	n	%	n
♂1	13 ♂ Dl^{05151}/Dl* NO EV<br>X<br>5 ♀ry/ry	1	25	96.1	26	29	9	23.7	38	30	3	9.1	33	35	3	7.9	38
	2 ♂ Dl^{05151}/Dl* LEV<br>X<br>5 ♀ry/ry	0	16	100	16	7	8	53.3	15	10	0	0	10	10	3	23.1	13
♂2	1 ♂ Dl^{05151}/Dl* L+REV<br>X<br>5 ♀ry/ry	1	23	95.8	24	11	15	57.7	26	16	2	11.1	18	30	2	6.2	32
	2 ♂ Dl^{05151}/Dl* LEV<br>6 ♂ Dl^{05151}/Dl* REV<br>X<br>5 ♀ ry^{-}/ry^{-}	1	47	97.9	48	17	12	41.4	29	12	4	25	16	20	8	28.6	28
♂3	5 ♂ Dl^{05151}/Dl* LEV<br>X<br>5 ♀ ry^{-}/ry^{-}	3	46	93.9	49	18	17	48.6	35	25	2	7.4	27	25	8	24.2	33
♂4	1 ♂ Dl^{05151}/Dl* L+REV<br>X<br>5 ♀ ry^{-}/ry^{-}	3	41	93.2	44	18	11	37.9	29	30	4	11.8	34	15	2	11.8	17
	4 ♂ Dl^{05151}/Dl* L or REV<br>X<br>5 ♀ ry^{-}/ry^{-}	2	33	94.3	35	18	11	37.9	29	30	3	9.1	33	20	2	9.1	22
♂5	1 ♂ Dl^{05151}/Dl* L+REV<br>X<br>5 ♀ ry^{-}/ry^{-}	7	14	60.9	21	10	4	26.7	14	34	3	8.1	37	32	3	8.6	35
	3 ♂ Dl^{05151}/Dl* LEV<br>5 ♂ Dl^{05151}/Dl* REV<br>X<br>5 ♀ ry^{-}/ry^{-}	7	8	53.3	15	29	14	32.6	43	25	0	0	25	**24**	2	7.7	26
	**TOT**	**25**	**253**	**91**	**278**	**157**	**101**	**39**	**258**	**212**	**21**	**9**	**233**	**211**	**33**	**13.5**	**244**
♂1	10 ♀ Dl^{05151}/Dl* L+R EV<br>X<br>5 ♂ ry^{-}/ry^{-}	0	32	100	32	4	27	87.1	31	30	12	28.6	42	11	5	31.2	16
♂2	10 ♀ Dl^{05151}/Dl* L+R EV<br>X<br>5 ♂ ry^{-}/ry^{-}	0	23	100	23	8	17	68	25	19	3	14.3	21	27	10	27	37
♂3	7 ♀ Dl^{05151}/Dl* L+R EV<br>X<br>5 ♂ ry^{-}/ry^{-}	1	37	97.4	38	6	49	89.1	55	23	14	37.8	37	34	15	30.6	49
♂4	10 ♀ Dl^{05151}/Dl* L+R EV<br>X<br>5 ♂ ry^{-}/ry^{-}	0	35	100	35	32	10	23.8	42	23	8	25.8	31	11	8	42.1	19
♂5	7 ♀ Dl^{05151}/Dl* L+R EV<br>X<br>5 ♂ ry^{-}/ry^{-}	0	29		29	6	19	76	25	20	4	16.7	24	20	4	16.7	24
	**TOT**	**1**	**156**	**99**	**157**	**56**	**122**	**68.5**	**178**	**115**	**41**	**26.4**	**155**	**103**	**42**	**28.9**	**145**
