# Supplementary material for: A new paramutation-like example at the Delta gene of Drosophila
Source: PLoS One. 2017 Mar 29;12(3):e0172780. doi: 10.1371/journal.pone.0172780 (PMC5371283; doi:10.1371/journal.pone.0172780)
Supplement: S3 Table — Females and males carrying the Dl05151 in heterozygosis with the TM3 or TM2 balancers were outcrossed to ry-/ry- and very few defective wings were found in the progenies containing the balancer chromosomes. WT: wild-type. D: dots. EV: extra-veins. TOT: total number of flies. (PDF) [file pone.0172780.s007.pdf]

PARENTS	Dl^{05151}/ry$^-$										TM3 or TM2/ry$^-$									
	♀♀					♂♂					♀♀					♂♂				
	WT	D	EV		TOT	WT	D	EV		TOT	WT	D	EV		TOT	WT	D	EV		TOT
	n	n	n	%	n	n	n	n	%	n	n	n	n	%	n	n	n	n	%	n
♀ Dl^{05151}/TM3 X ♂ ry$^-$/ry$^-$	2	0	45	96	47	9	0	32	78	41	30	1	0	0	31	21	1	0	0	22
♀ ry$^-$/ry$^-$ X ♂ Dl^{05151}/TM3	3	0	65	96	68	10	1	46	80	57	29	1	0	0	30	23	1	0	0	24
♀ Dl^{05151}/TM2 X ♂ ry$^-$/ry$^-$	2	0	48	96	50	3	0	49	94	52	25	0	0	0	25	34	1	1	3	36
♀ ry$^-$/ry$^-$ X ♂ Dl^{05151}/TM2	3	0	35	92	38	8	0	33	80	41	47	2	2	4	51	38	1	0	0	39
